# Explaining compound activity predictions with a substructure-aware loss for graph neural networks

**DOI:** 10.1186/s13321-023-00733-9

**Published:** 2023-07-25

**Authors:** Kenza Amara, Raquel Rodríguez-Pérez, José Jiménez-Luna

**Affiliations:** 1 Microsoft Research AI4Science, 21 Station Rd., Cambridge, CB1 2FB UK; 2 Novartis Institutes for Biomedical Research, Novartis Campus, 4002 Basel, Switzerland; 3 Department of Computer Science, ETH Zurich, Andreasstrasse 5, 8050 Zurich, Switzerland

**Keywords:** Explainable AI, Model interpretation, Graph neural networks, Benchmark, Activity predictions, QSAR, Lead optimization, Drug discovery

## Abstract

Explainable machine learning is increasingly used in drug discovery to help rationalize compound property predictions. Feature attribution techniques are popular choices to identify which molecular substructures are responsible for a predicted property change. However, established molecular feature attribution methods have so far displayed low performance for popular deep learning algorithms such as graph neural networks (GNNs), especially when compared with simpler modeling alternatives such as random forests coupled with atom masking. To mitigate this problem, a modification of the regression objective for GNNs is proposed to specifically account for common core structures between pairs of molecules. The presented approach shows higher accuracy on a recently-proposed explainability benchmark. This methodology has the potential to assist with model explainability in drug discovery pipelines, particularly in lead optimization efforts where specific chemical series are investigated.

**Supplementary Information:**

The online version contains supplementary material available at 10.1186/s13321-023-00733-9.

## Introduction

Drug discovery is one of the many fields where deep learning techniques have found extensive applicability in the last few years [1]. While the history behind traditional machine learning (ML) in cheminformatics can be traced as far back to the 1960 s [2, 3], some recently-adopted deep learning paradigms have become increasingly popular across many tasks (e.g., *de novo* molecular design, synthesis prediction). Specifically, *in silico* molecular property prediction (also commonly referred to as quantitative structure–property relationship modeling) is a central challenge in drug discovery where graph neural networks (GNNs) [4] have shown promising performance. Among the many factors that contributed to the popularity of GNNs in chemistry and other areas, we can highlight their suitability to naturally perform automatic feature extraction on arbitrarily-sized graphs and their scalability to existing commodity hardware. In chemistry, GNNs can take advantage of the natural description of molecules as graphs, where atoms and bonds can be represented as nodes and edges, respectively. Recent applications of GNN for molecular property prediction include *in vivo* brain penetration [5], *in vitro* intrinsic clearance [6], among others [7–9].

However, the popularity of GNNs has also been accompanied by an increasing need for explainability [10–18], as these models have been notoriously known for their black-box character. Towards this goal, explainable artificial intelligence techniques, such as feature attribution analyses, have become relevant tools. These analyses provide an importance value for every input feature, atom or bond in a molecular graph. Such importance values are often visualized through atom or bond coloring, where the structural patterns that drive a prediction are highlighted on top of the two-dimensional molecular representation of the compound of interest [19].

Towards disentangling what structural patterns are exploited by GNNs in compound property predictions, a variety of feature attribution techniques have been previously reported in the literature [20]. Importantly, many research efforts have focused on benchmarking feature attribution techniques, exploring their consistency and quality in atom coloring, and providing recommendations [21–24]. In particular, one such study proposed a quantitative benchmark based on publicly-available activity data for congeneric series and evaluated the performance of several GNN architectures and feature attribution techniques [25]. Therein, it was shown that GNNs did exhibit some degree of accordance with the predefined colors of the benchmark, but their explainability performance fell markedly behind simpler techniques such as atom masking [26] in combination with more traditional machine learning methods such as random forests (RF).

In order to mitigate this issue, in this paper we propose a training loss modification for GNNs that improves explainability performance on the aforementioned benchmark. Our method takes advantage of the fact that lead optimization efforts focus on specific compound series, where molecules share structural cores (i.e., scaffolds). The explicit consideration of the molecular scaffold formalism can be leveraged to appropriately assign importance of the uncommon substructures responsible for a property change during model training. We show that the proposed approach is beneficial towards closing the explainability performance gap previously reported between GNNs and other classical methods. The architecture is inspired by recent work on molecular representation learning based on reaction data that explicitly encourage the similarity of reactants and reagents in embedding space [27]. To foster reproducibility, all code and data are made available through a permissive open-source license.

## Materials and methods

### Benchmark data

**Fig. 1:**
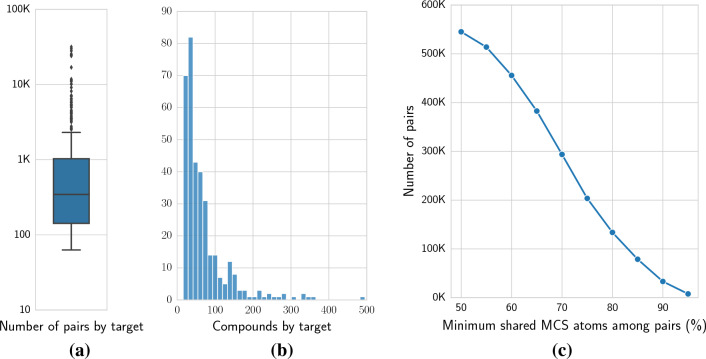
Benchmark descriptive analyses. Reported are **a** the distribution of number of pairs per protein target, **b** the number of compounds per protein target, and **c** the number of compound pairs considered at varying scaffold size (different thresholds of minimum shared MCS among pairs)

#### Molecular scaffolds

A scaffold is defined as the core of the molecule where one or several functional groups can be attached. Molecular scaffolds constitute the basis of structure-activity relationships (SAR) analyses. Even though ligand-based drug discovery does not explicitly cover the study of specific interactions with the protein target, it is well-suited for human interpretability. In fact, numerous ligand-based drug discovery efforts focus on these SAR analyses e.g., matched molecular pairs (MMPs), specially in lead optimization [28, 29]. Herein, the maximum common substructure (MCS) formalism was used to define a molecular scaffold [30] between pairs of compounds binding to a specific target. To consider that two compounds share a molecular scaffold, such common part should encompass a minimum fraction of their structure. Taking this into consideration and in line with previous work, different thresholds of minimum shared substructures were examined [25]. For the development and evaluation of our methodology, MCS pairs were computed using the FMCS [31] algorithm, as available in the RDKit *rdFMCS* module [32].

#### Data preparation

The benchmark data from a recently proposed study on feature attribution [25] was used, which consisted of 723 protein targets with associated small molecule activity data (half maximal inhibitory concentration, IC$_{50}$). A negative logarithmic transformation was applied to IC_50_ concentrations to obtain *p*IC_50_ values. The dataset was initially constructed using the BindingDB protein-ligand validation sets [33], which contains binding affinities for a large number of targets and across different molecular scaffolds. In said data set, ground-truth atom-level feature attribution labels were determined via the concept of activity cliffs [34–39]. Specifically, these were defined as pairs of compounds in one or multiple congeneric series sharing a molecular scaffold and with at least 1 log unit activity difference. Compounds for each protein target were randomly divided into training (80%) and test (20%) sets. Only protein targets with at least 50 compound pairs in the training set were kept. To avoid data leakage, the same compound was not allowed to be present in different pairs in training and test sets, resulting in a final selection of 350 protein targets. Figure 1 shows the distribution of the number of pairs and compounds per target at the minimum considered MCS threshold of 50%, as well as the number of pairs sharing molecular scaffolds at different minimum thresholds.

### Models and feature attribution techniques

#### Models

Message-passing GNN [40] models were trained to predict compound activity against all available protein targets. In most molecular property prediction scenarios, these are models $f \in {\mathcal {F}}$ that map molecular graphs to real values $f: {\mathcal {G}}({\mathcal {V}}, {\mathcal {E}}) \rightarrow {\mathbb {R}}$, with $v \in {\mathcal {V}}, e \in {\mathcal {E}}$ representing atoms and bonds, respectively. They do so by iteratively learning and updating internal node latent representations using the information from neighboring atom and bond latent spaces (for a more comprehensive description a canonical reference is provided in Gilmer et al. [4]). In this work GNNs were optimized to minimize at least one of the following loss functions: (i) mean squared error (MSE) between observed and predicted binding affinities (in logarithmic scale), (ii) a relative potency loss computed on pairs of related compounds, hereby referred to as activity cliff (AC) loss, and (iii) the proposed uncommon node loss (UCN). Both AC and UCN losses were considered on top of the standard MSE loss with a fixed weighting term (see "Substructure-aware loss" section). As a control, random forest (RF) models trained with extended-connectivity fingerprints (ECFP4) were also considered. Additional details regarding neural network hyperparameters, featurization, and optimization details are provided in Additional file 1: Section 5.

#### Feature attribution techniques

In the context of this work, feature attribution techniques are functions that take a molecular graph and a trained property model and produce a real number (i.e., a coloring) for each atom in the graph. Such values represent atomic importance for the prediction. $e: ({\mathcal {G}}, {\mathcal {F}}) \rightarrow {\mathbb {R}}^{{\mathcal {V}}}$. Following previous benchmarking work [20, 25], a variety of feature attribution methods that enable the estimation of positive and negative atom contributions were investigated. Class Activation Maps (CAM) [41] and gradient-based methods, namely GradInput [42], Integrated Gradients [43], and Grad-CAM [44] were utilized. Additionally, other perturbation-based approaches such as node masking, where the contribution of each atom is determined as the difference in prediction upon its artificial modification, were considered. For the presented GNN models, node masking iteratively set node features to zero. For RF models, each atom was assigned an atom type that was not present in the benchmark sets, and molecular features re-calculated [26]. Section 6 in Additional file 1 reports additional technical details and explanations on each of the feature attribution methods used as well as their chosen hyperparameters.

### Substructure-aware loss

**Fig. 2:**
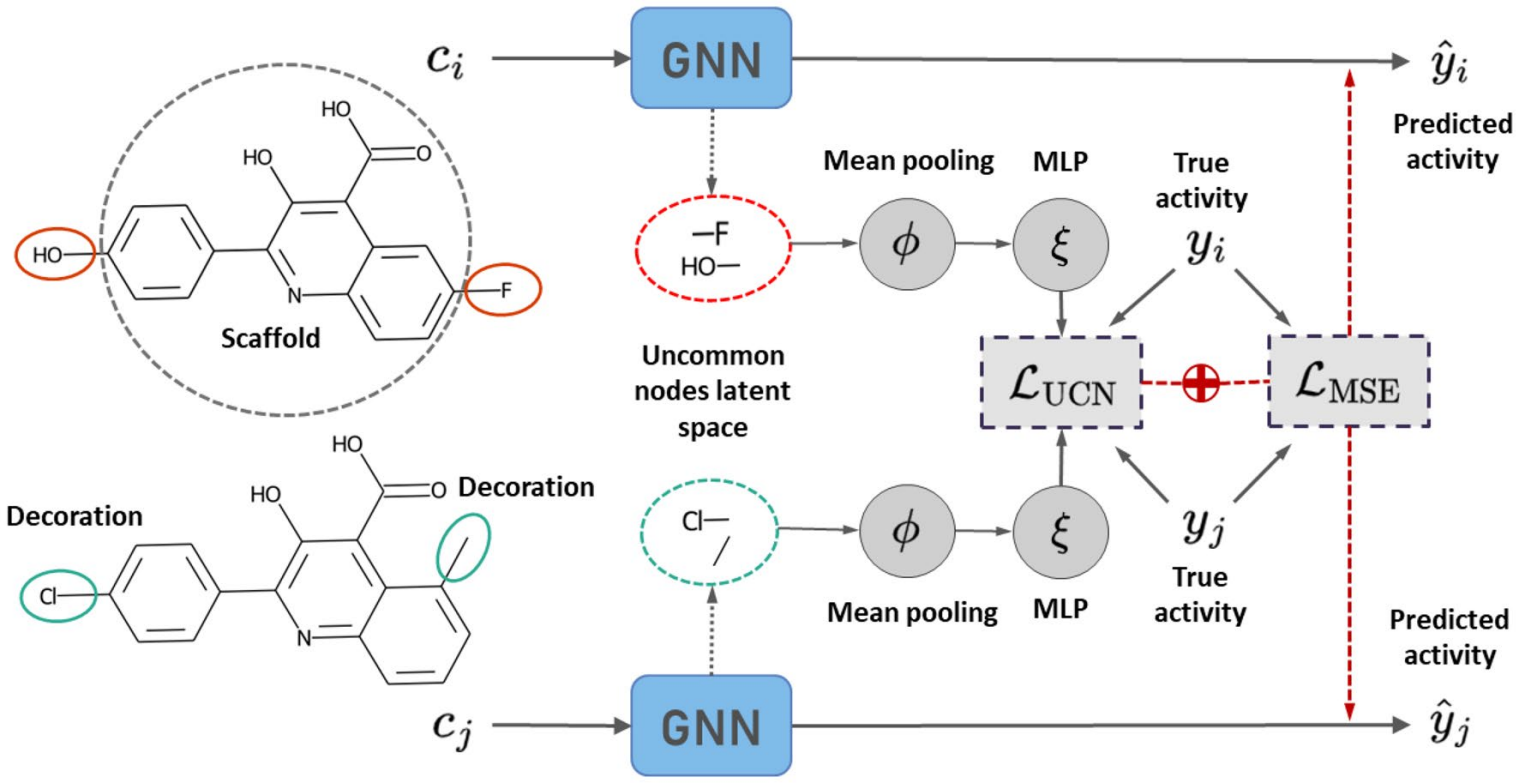
Schema of the proposed UCN loss. Two compounds sharing a scaffold are sampled from the training set, and their atom latent spaces computed via a forward pass of a GNN model. The uncommon latent nodes are used for the loss computation, targeting the activity difference between the compound pairs. In the illustrated example, the compound pair is composed by $c_{i}$ and $c_{j}$, with a large MCS and two substitution sites, highlighted in red for $c_{i}$ and green for $c_{j}$. Substituents (or decorations) differ for both compounds, and correspond to the uncommon nodes in the latent space

A supervised learning problem was considered where a GNN model was trained to predict compound activity against a specific protein target. Motivated by the fact that several drug discovery efforts tend to focus on congeneric series (*e.g.,* lead optimization), we propose a loss that focuses on the uncommon structural motifs between ligand pairs. A schematic representation of this procedure is provided in Fig. 2. During training, compound pairs with a common scaffold are sampled and the difference in predicted activity is attributed to the uncommon node latent spaces. For each pair *k* of compounds *i*, *j*, with corresponding molecular graphs $c_i, c_j \in C$ and experimental activities $y_i, y_j \in {\mathbb {R}}$, the proposed uncommon node loss is computed as:

1 $$\begin{aligned} {\mathcal {L}}_{\text{UCN}}\left( c_i, c_j, k\right) := \left\Vert \left( \xi \left( \phi \left( M_i^k \left( \varvec{h}_i \right) \right) \right) - \xi \left( \phi \left( M_j^k \left( \varvec{h}_j \right) \right) \right) \right) - (y_i - y_j) \right\Vert ^2, \end{aligned}$$

where $\varvec{h}_i \in {\mathbb {R}}^{N_i \times d}$ is the latent node representation of compound $c_i$, $M^{k}_i: {\mathbb {R}}^{N_i\times d} \rightarrow {\mathbb {R}}^{n_i\times d}$ is a masking function over nodes that retrieves those uncommon for compound *i* in the context of pair *k*, $\phi : {\mathbb {R}}^{n\times d} \rightarrow {\mathbb {R}}^{d}$ is a mean readout function over nodes, $\xi : {\mathbb {R}}^{d} \rightarrow {\mathbb {R}}$ is a multilayer perceptron with linear activation, and $\left\Vert \cdot \right\Vert$ is the vector Frobenius norm. During model training, the UCN term was used alongside of a standard mean squared error (MSE) loss on the absolute predicted versus experimental binding affinities of pair *k*:

2 $$\begin{aligned} {\mathcal {L}}_{\text{MSE}}\left( c_i, c_j \right) := \left\Vert y_i - \hat{y}_i\right\Vert ^2 + \left\Vert y_j - \hat{y}_j\right\Vert ^2, \end{aligned}$$

where $\hat{y}_i$ is an absolute activity prediction output that aggregates over all available nodes in each pair (*i.e.,* both common and uncommon). Since sampling compound pairs results in an augmented data set that could artificially boost performance, additional models were trained to minimize a relative potency loss:

3 $$\begin{aligned} {\mathcal {L}}_{\text{AC}}\left( c_i, c_j \right) := \left\Vert \left( y_i - y_j\right) - \left( \hat{y}_i - \hat{y}_j\right) \right\Vert ^2. \end{aligned}$$

Specifically, the models considered in this study were trained to minimize either ${\mathcal {L}}_{\text{MSE}}$ or one of the two combinations ${\mathcal {L}}_\mathrm {MSE+AC}:= {\mathcal {L}}_{\text{MSE}} + \lambda {\mathcal {L}}_{\text{AC}}$, ${\mathcal {L}}_\mathrm {MSE+UCN}:= {\mathcal {L}}_{\text{MSE}} + \lambda {\mathcal {L}}_{\text{UCN}}$. For all training and testing purposes in this study we fix $\lambda =1$.

This loss function is specifically-designed to put more emphasis on the uncommon nodes causing the activity change during training. However, at inference time, the scaffold does not need to be predefined, *i.e.,* the model does not receive any information about common nodes. Therefore, the proposed architecture can be applied to compounds that do not have any analog in the training set (i.e., a new chemical series).

### Evaluation metrics

#### Predictive performance

Regression model performance against individual targets was evaluated with the root mean squared error (RMSE) and Pearson’s correlation coefficient (PCC) metrics. To aggregate results across all targets in the data set, both the unweighted (simple) and weighted average values were calculated. For the weighted average calculation, RMSE or PCC values were weighted by the number of compounds pairs in the test set of each target.

#### Explainability

The performance of the feature attribution methods was evaluated using *global direction* and *atom-level accuracy* metrics [25]. Global direction is a binary metric assessing whether average feature attribution across the uncommon nodes in a pair *k* of compounds preserves the direction of the activity difference. Assuming $\psi : C \rightarrow {\mathbb {R}}^{N\times d}$ is a feature attribution function that assigns a score to each node feature in an input graph, the metric for a single pair is computed as:

4 $$\begin{aligned} g_{\text{dir}}\left( c_i, c_j \right) = \mathbbm {1}\left[ {{\,\text{sign}\,}}{\left( \Phi \left( M_i^k\left( \psi \left( c_i \right) \right) \right) - \Phi \left( M_j^k\left( \psi \left( c_j \right) \right) \right) \right) } = {{\,\text{sign}\,}}{\left( y_i - y_j \right) } \right] , \end{aligned}$$

where $\Phi : {\mathbb {R}}^{N\times d} \rightarrow {\mathbb {R}}$ is a mean aggregator over nodes and features. The score is averaged over all pairs in the benchmark test sets.

Atom-level accuracy, also hereby referred to as *color agreement*, measures whether the feature attribution assigned to a node has the same sign as the experimental activity difference of the compound pair (ground truth). In previous work, ground-truth atom attribution labels were obtained by assuming that the structural changes between a pair of compounds were responsible for the observed potency changes [25]. Therefore, structural parts in the most potent compound of the pair were assigned a positive feature attribution, and vice versa. For every atom in a compound with corresponding molecular graph $c_i$ with $m_i$ common atoms in pair *k*, and with ground truth atom color $\varvec{t}_{i}^k \in \left\{ -1, 1 \right\} ^ {m_i}$, the (vector-valued) metric is defined as:

5 $$\begin{aligned} g_{\text{atom}}\left( c_i \right) := \mathbbm {1}_{m_i}\left[ {{\,\text{sign}\,}}{\left( \eta \left( M_i^k\left( \psi \left( c_i \right) \right) \right) \right) } = \varvec{t}_i^k \right] , \end{aligned}$$

where $\eta : C \rightarrow {\mathbb {R}}^N$ is a mean aggregation function over features and $\mathbbm {1}_{m_i}$ is an indicator vector with $m_i$ binary entries. The mean value $\bar{g}_{\text{atom}}$ is then used as a summary of the color accuracy for compound $c_i$.

Jiménez-Luna et al. [25] noted that the ground-truth colors assigned by $g_{\text{atom}}$ can be ill-defined for a compound, since they are dependent on the other compound in the pair (*i.e.,* the assigned colors to one compound could either be positive or negative depending on the specific comparison). In contrast, $g_{\text{dir}}$ does not suffer from this problem. For this reason, the analyses reported here focus on the $g_{\text{dir}}$ evaluation metric and, for completeness, $g_{\text{atom}}$ results are reported in Section 4 of Additional file 1.

## Results and discussion

ML models were generated to predict compound potency against 350 protein targets. Message-passing GNNs were trained to minimize different loss functions, including the standard MSE, its linear combination with relative (AC), and the uncommon node (UCN) losses. Moreover, RF models were built for comparison. First, prediction performance was assessed for all GNN and RF models. Next, model explainability was benchmarked and the influence of the UCN loss analyzed for individual targets. Potential factors influencing explainability were analyzed. Finally, potential applications of the proposed UCN loss and feature attribution methods are shown.

### Predictive performance

There is a known trade-off between model interpretability and accuracy [45]. Model explanations could be incorrect (feature attributions could be inaccurate) even if the ML model predicts the correct direction of potency change. Moreover, only explanations from well-performing methods can be used to assist in drug design. Therefore, prediction performance was evaluated for all GNN and RF models. Table 1 reports the simple and weighted average values for root mean squared error (RMSE) and Pearson’s correlation coefficient (PCC) metrics. Results are shown for GNNs built with different loss functions, *i.e.,* solely MSE loss (${\mathcal {L}}_\text{MSE}$), MSE in combination with AC (${\mathcal {L}}_\mathrm {MSE+AC}$) or UCN losses (${\mathcal {L}}_\mathrm {MSE+UCN}$), and RF. Average RMSE values across all targets ranged from 0.31 (GNN with ${\mathcal {L}}_\mathrm {MSE+AC}$) to 0.47 (GNN with ${\mathcal {L}}_\mathrm {MSE+UCN}$). Average correlation between predicted and experimental potency values ranged from 0.84 (GNN with ${\mathcal {L}}_\mathrm {MSE+UCN}$) to 0.95 (RF). Weighted average RMSE and PCC values were also calculated, where the results for each target were weighted by the number of compounds in the test set. The smallest and largest weighted average RMSE were 0.24 (GNN with ${\mathcal {L}}_\mathrm {MSE+AC}$) and 0.37 (${\mathcal {L}}_\mathrm {MSE+UCN}$). In addition, weighted average correlation values were between 0.93 (GNN with ${\mathcal {L}}_\mathrm {MSE+UCN}$) and 0.96 (rest of the methods). Only minor differences favouring the ${\mathcal {L}}_\mathrm {MSE+AC}$ loss for RMSE values were observed, with most results lying within one standard deviation of each other. Interestingly, the simple and the weighted average version of the metrics differed more for GNN models. These results suggest that GNN predictive ability might be more affected by the size of the training data set (which in this case was correlated with the test set size) than RF models. To complement these analyses, relative performance between RF and GNN models at different training set sizes are reported in Additional file 1: Section 1.

Even though the UCN loss function utilizes the information of scaffolds and uncommon nodes (substitution sites) during model training, scaffolds do not need to be defined at inference time. This makes the UCN loss also applicable to explain compound predictions for new chemical series, which is the application shown herein. Higher performance values would be expected if compound analogs were present in the training set [46].

**Table 1:** Test set predictive performance

	Avg. RMSE	W. Avg. RMSE	Avg. PCC	W. Avg. PCC
RF	$0.35\, (\pm 0.11)$	$0.30\, (\pm 0.08)$	$0.95\, (\pm 0.07)$	$0.96\, (\pm 0.04)$
GNN ${\mathcal {L}}_{\text{MSE}}$	$0.34\, (\pm 0.23)$	$0.25\, (\pm 0.13)$	$0.89\, (\pm 0.23)$	$0.96\, (\pm 0.08)$
GNN ${\mathcal {L}}_{\mathrm {MSE+AC}}$	$0.31\, (\pm 0.24)$	$0.24\, (\pm 0.13)$	$0.89\, (\pm 0.23)$	$0.96\, (\pm 0.07)$
GNN ${\mathcal {L}}_{\mathrm {MSE+UCN}}$	$0.47\, (\pm 0.28)$	$0.37\, (\pm 0.14)$	$0.84\, (\pm 0.24)$	$0.93\, (\pm 0.08)$

Reported are the average (Avg.) and weighted average (W. Avg., over number of compounds per target) of root mean squared error (RMSE) and Pearson’s correlation coefficient (PCC) values (± 1 standard deviation)

**Fig. 3:**
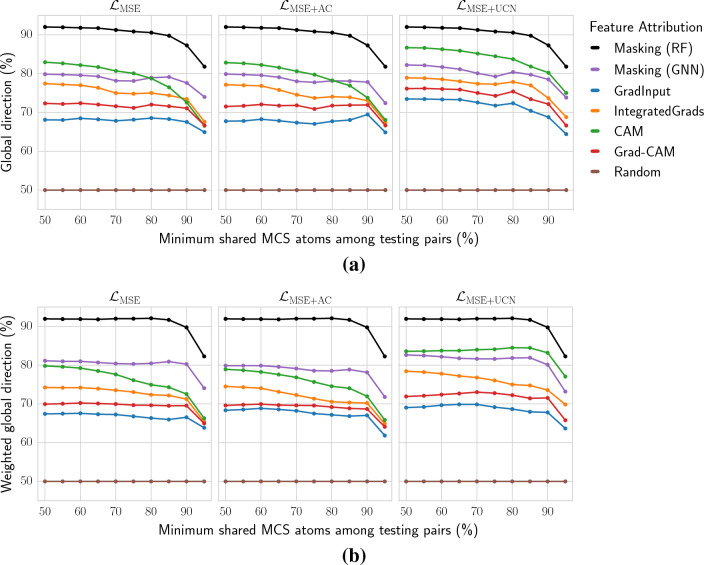
Global direction at varying scaffold size and across feature attribution methods. **a** Global direction and **b**, weighted global direction values are reported at different thresholds of minimum shared MACS among testing pairs (%). In **b**, global direction is weighted by the number of pairs per each target. Results are shown for three loss functions, i.e. ${\mathcal {L}}_{\text{MSE}}$ (left panel), ${\mathcal {L}}_{\mathrm {MSE+AC}}$ (middle panel), and ${\mathcal {L}}_{\mathrm {MSE+UCN}}$ (right panel). Colors report different feature attribution methods, five for GNN models and atom masking for RF models. Since the three losses functions are only applied to GNN models, RF results are equivalent in the three panels. An additional random feature attribution line is included as a baseline

### Explainability evaluation at varying scaffold size

Explainability was primarily evaluated using the global direction score, which focuses on the uncommon nodes for a compound pair and assesses whether the direction of the activity difference is preserved. Global direction values were calculated at varying MCS thresholds among compound pairs. Figure 3 shows the global direction values for all test pairs and targets considered in the study. Many feature attribution methods applied to GNNs with the proposed UCN objective (${\mathcal {L}}_{\mathrm {MSE+UCN}}$) exhibited larger global direction values over the absolute MSE (${\mathcal {L}}_{\text{MSE}}$) and relative MSE (${\mathcal {L}}_{\mathrm {MSE+AC}}$) losses. Improvements were observed for most methods, but were more pronounced for CAM, Grad-CAM, and GradInput. Additionally, the GNN-based masking method also exhibited a slight performance increase. Most importantly, this explainability improvement held across different thresholds of minimum MCS between pairs. Figure 3b reports the results with the weighted color direction metric, where similar conclusions can be drawn. In this case, Integrated Gradients showed larger improvements compared to the non-weighted analyses. Despite the global direction improvement for GNNs with ${\mathcal {L}}_{\mathrm {MSE+UCN}}$ loss, RF models with an atom masking approach achieved larger values. Among the GNN methods, CAM and masking approaches provided top-performing global direction results. Global direction values were overall stable across different scaffold size. Only when the uncommon structural parts in compound pairs were small (MCS thresholds > 85–90%), global direction values significantly decreased for all methods. Additional file 1: Section 2 reports absolute differences in global direction across the different GNN loss functions considered.

**Fig. 4:**
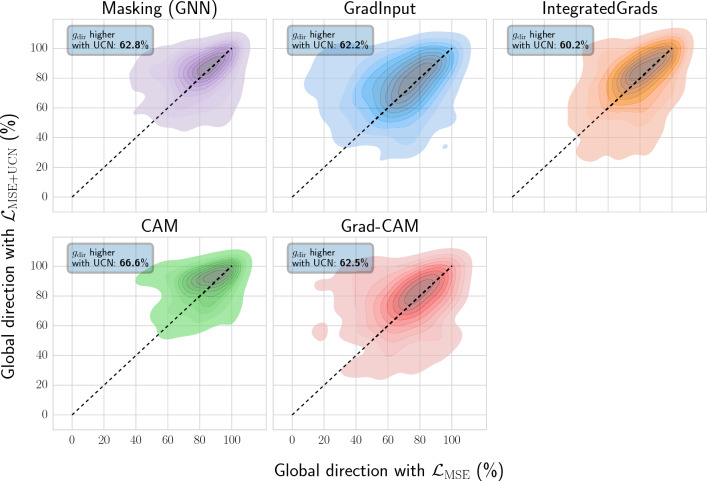
Per-target comparison of global direction values. The two-dimensional kernel density plot shows the target-specific global direction values with ${\mathcal {L}}_{\texttt {MSE}}$ (x-axis) and ${\mathcal {L}}_{\texttt {MSE+UCN}}$ (y-axis) loss functions. The text-box reports the percentage of protein targets for which global direction ($g_\text{dir}$) was larger with ${\mathcal {L}}_{\texttt {MSE+UCN}}$ loss. Compound pairs considered at the minimum 50% MCS threshold

### Explainablity for individual protein targets

In the previous section, explainability methods were benchmarked using the average global direction across all targets. Nevertheless, for specific protein targets, the best explainability method might differ. To evaluate how often this is the case, global direction with ${\mathcal {L}}_{\text{MSE}}$ and ${\mathcal {L}}_{\mathrm {MSE+UCN}}$ loss functions were compared on a per-target basis (Fig. 4). Global direction values were higher for 60–66% of the targets when including the UCN loss. Additionally, most feature attribution methods showed improvements with the UCN loss, with CAM exhibiting the largest improvements ($66\%$). Additional plots and analyses can be found in Additional file 1: Section 3, where CAM approached the performance of RF masking when evaluated on the training sets. Additional file 1: Section 4 reports results with color agreement as an alternative metric. In that case, the UCN loss produced an improvement for several of the feature attribution methods in both training and test sets, albeit the advantage was less pronounced than with the global direction metric.

**Fig. 5:**
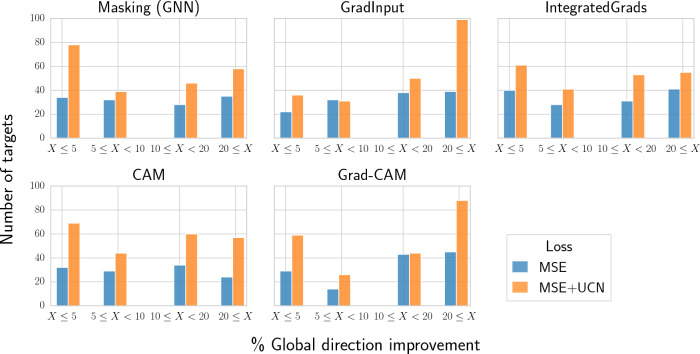
Protein targets with global direction improvements. Reported are the number of targets (y-axis) displaying a given improvement of the global direction metric $g_{\text{dir}}$ using the proposed $\mathcal {L_\mathrm {MSE+UCN}}$ loss compared to ${\mathcal {L}}_{MSE}$ (x-axis). Global direction improvements were binned into $\le$5%, between 5 and 10%, between 10 and 20%, and $\ge$20% thresholds. Colors indicate the loss function utilized during GNN training (${\mathcal {L}}_{MSE}$, blue; $\mathcal {L_\mathrm {MSE+UCN}}$, orange). A minimum threshold of 50% MCS was considered for this analysis

Figure 5 reports the number of targets for which the addition of the UCN loss term led to a negligible ($\le$5%), small (between 5% and 10%), medium (between 10% and 20%), or large ($\ge$20%) global direction improvement. Results indicate that GNNs with ${\mathcal {L}}_{\texttt {MSE+UCN}}$ loss led to larger global direction values for the same or higher number of targets than GNNs with the standard ${\mathcal {L}}_{\texttt {MSE}}$ loss. Interestingly, differences across loss functions became larger when considering targets with medium to large global direction improvements in their explanations. CAM, GradInput, and Grad-CAM showed the largest benefit of UCN loss inclusion, with many targets having global direction improvements higher than 20% (133 for Grad-CAM, 138 for GradInput, and 81 for CAM).

### Potential factors influencing explainability

**Fig. 6:**
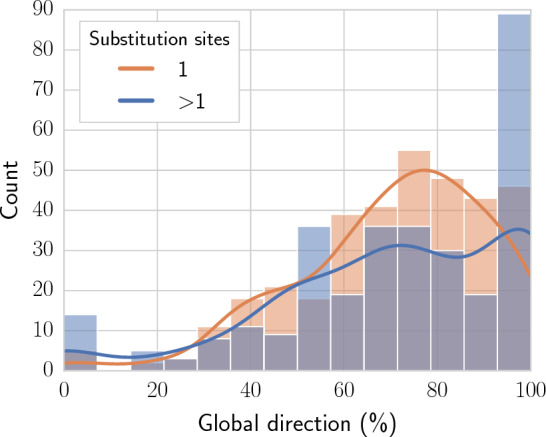
Effect of the number of substitution sites on the global direction metric. Global direction (x-axis) is reported for compound pairs with a single (orange) or multiple (blue) substitution sites. For the derivation of compound pairs, a minimum 50% MCS threshold was set

**Fig. 7:**
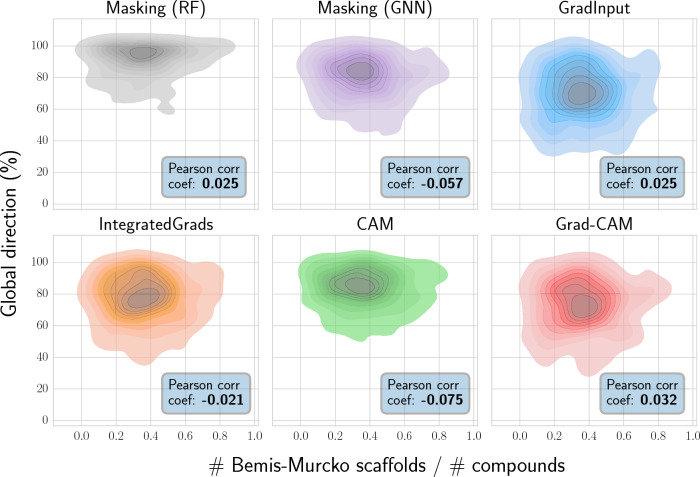
Effect of structural diversity on global direction. Reported are the per-target chemical diversity and global direction values per each protein target. Results reported for the minimum 50% MCS threshold

As a way of elucidating which factors contribute to a successful feature attribution assignment, the benchmark was extended to evaluate whether $g_{\text{dir}}$ is affected by (i) the number of substituent sites in the compound pair [37], or (ii) the chemical diversity within the ligands for each target. Figure 6 reports the global direction values for compound pairs that differ by one or at least two substitution sites. Results suggested that feature attribution methods did not showcase an overall higher performance for compounds pairs that differ in a single substitution site. Additionally, chemical diversity was estimated via the Bemis-Murcko scaffold [47] formalism (Fig. 7). In more detail, chemical diversity was defined as the total number of scaffolds divided by the number of compounds available for each target. Apart from a slightly higher concentration of targets around areas where both the number of scaffolds is low and $g_{\text{dir}}$ is high, no significant correlation between these values was observed.

### Exemplary applications

**Fig. 8:**
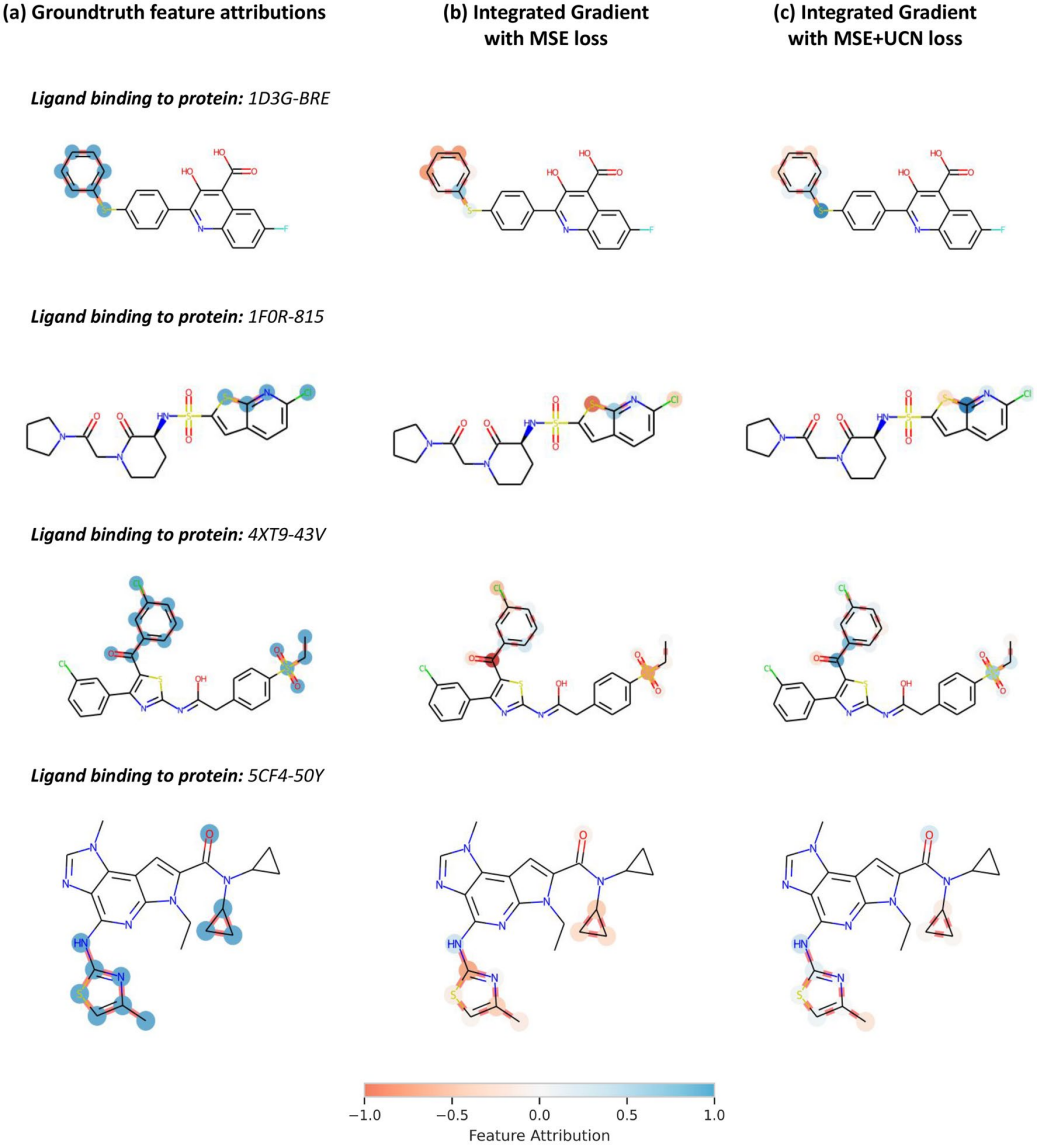
Exemplary explanations for test set molecules. **a** Ground-truth feature attributions from the benchmark, **b** Integrated Gradients with MSE loss, and **c** with MSE+UCN loss results are reported with a coloring scheme. In the first two examples (PDB Ids. 1D3G, 1F0R), compounds had a single substitution site. The model trained with the simpler MSE loss failed to correctly capture the direction of the activity change (indicated by the ground-truth). The third and fourth examples (PDB Ids. 4XT9, 5CF4) constitute compounds from pairs that differed in multiple substitution sites. Feature attribution methods are also be applicable. Both the UCN and the simple MSE loss provide similar colors for all but one site

**Fig. 9:**
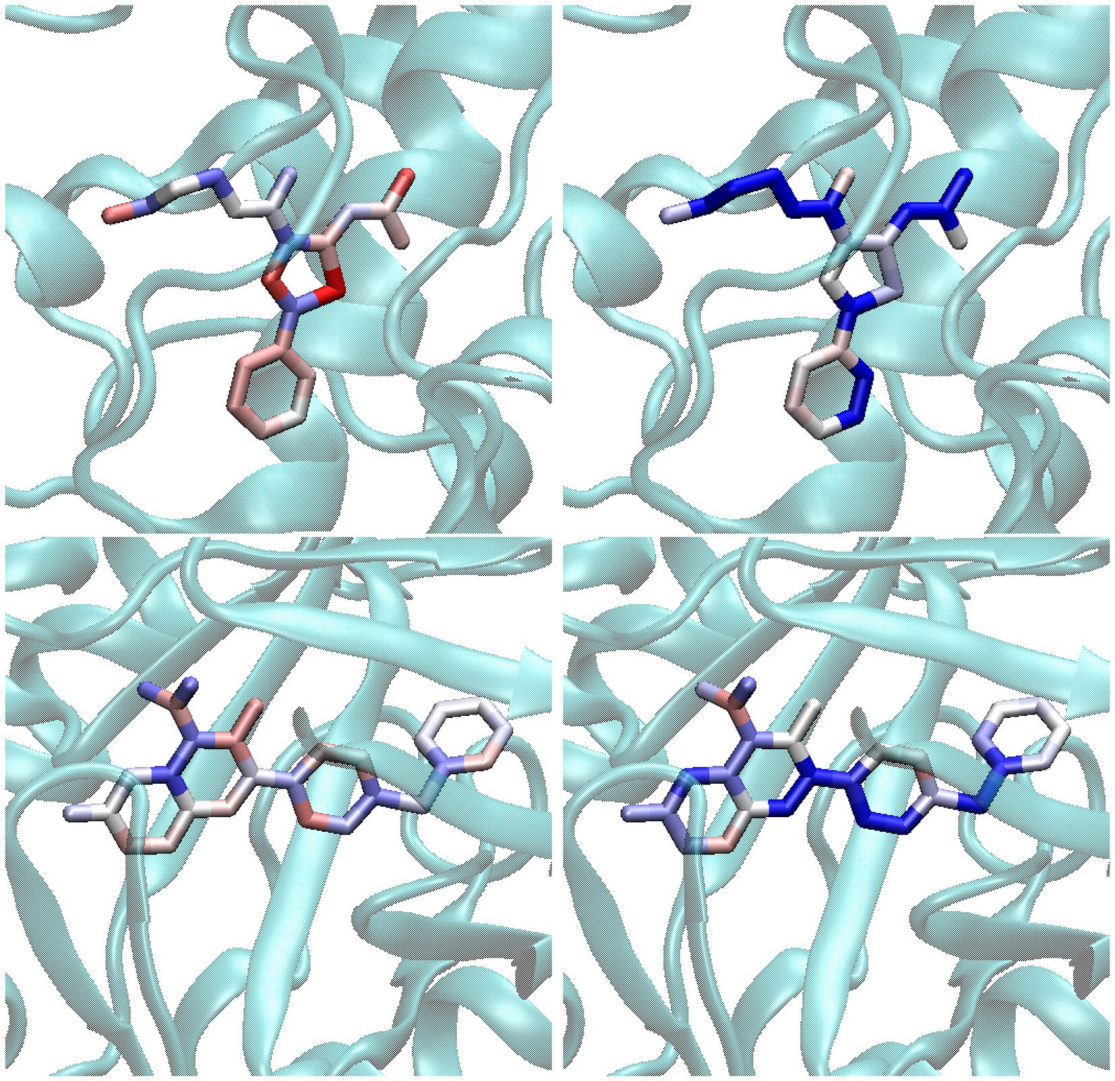
Mapping of feature attributions for visualizations after docking. Feature attribution values were mapped to two compound structures in the context of their binding receptors (PDB Ids. 2YDK and 1D3G). Attributions computed using Integrated Gradients (top row, PDB Id. 2YDK) and GradInput (bottom row, PDB Id. 1D3G), and using the ${\mathcal {L}}_{\text{MSE}}$ (left column) to ${\mathcal {L}}_{\mathrm {MSE+UCN}}$ (right column) losses, are reported

The current set-up with compound analogs that differ at a single or multiple substitution sites facilitates a systematic explainability method benchmark due to the definition of a ‘ground truth’ based on potency differences. Nevertheless, when using this method in practice, more opportunities and potential applications exist. Actually, this GNN explainability method can be applied to any molecule to obtain attributions for all atoms. Therefore, it is possible to estimate which substitution site is more responsible for predicted activity.

As a way of exemplifying how the proposed methodology can be used in practice, Fig. 8 reports feature attributions for two active compounds against human dihydroorotate deydrogenase (PDB Id. 1D3G) and coagulation factor Xa (PDB Id. 1F0R). The first column (**a**) reports the ground-truth atomic attribution labels, assigned from the comparison to other analog pairs, while (**b**) and (**c**) contain attributions computed via the Integrated Gradients method with either the MSE or the UCN loss, respectively. Interestingly, the proposed UCN loss function yielded better explanations than the simpler MSE loss. For instance, for the ligand binding to protein 1F0R, the ground-truth attribution labels were marked as positive, whereas the average attributions obtained with the MSE and MSE+UCN losses were $-0.27$ and $+0.39$, respectively. These results indicate that UCN loss correctly assessed the direction of the attribution.

As also shown in Fig. 8, compounds with differences in multiple substitution sites can be compared. One ligand of nuclear receptor ROR$\gamma$ (PDB Id. 4XT9) and one of Tyrosine-protein kinase JAK2 (PDB Id. 5CF4) are shown. In these examples, attributions assigned to the specific uncommon motifs are similar, but the UCN loss distinguishes one of those as responsible for the predicted activity change. Therefore, the method can also help generating hypotheses about which substitution sites are driving activity predictions. Computed attributions for all molecules and methods considered in this study are also made available through the accompanying code repository to this work.

While only the ligand-based paradigm is considered in this study, structural motifs that have been assigned a high importance by the GNN explainability method could be examined after docking. Figure 9 shows feature attributions for two compounds in the context of their binding receptors (PDB Ids. 2YDK and 1D3G, with $p\text{IC}_{50}$ values of 7 and 7.74 units, in the top and bottom rows respectively). Poses were computed using the Vina software package [48]. In these examples, GNN models trained with the UCN loss (right column) gave higher attribution to structures that are responsible for key interactions. In the case for the ligand selected for Serine/Threonine-protein kinase CHK1 (PDB Id. 2YDK), only the model trained with the additional UCN loss was able to identify some of the key interactions, namely hydrogen bonds with residues SER193, ILE131 and THR170 and a $\pi$-cation interaction with ARG129. As for the ligand selected for Dihydroorotate dehydrogenase (PDB Id. 1D3G), one of the central aromatic rings was correctly identified as engaging in a parallel $\pi$-stacking interaction with TYR208. The ring on the right-hand side leads to better coverage of the binding pocket through additional hydrophobic interactions, which is contradictorily predicted as a negative contribution by the model with MSE loss.

## Conclusions

In this study, we explored and quantitatively evaluated how the explainability of GNNs can be improved in the context of drug discovery. Specifically, a novel substructure-aware loss was proposed to improve GNNs’ explainability for congeneric series data. This modified loss function was evaluated on a previously-reported benchmark for molecular ML explainability and it was observed that most GNN-based feature attribution techniques markedly benefited from its usage. Global direction values were used to evaluate compound explanations. Our results showed that the average global direction as well as the percentage of targets with global direction improvements were superior with the consideration of the UCN loss during GNN training. Specifically, a 66% and 63% of the targets improved global direction scores for CAM and GNN masking, respectively, which were identified as the best-performing GNN feature attribution methods. Moreover, when explaining activity predictions for a specific target protein, large global direction improvements were more likely with the newly proposed loss function. However, despite the observed superiority of the substructure-aware loss in GNN-based feature attribution methods, the RF models coupled with an atom masking approach still remained the best approach for explainability in the benchmark [26]. Nevertheless, the feature attribution performance gap between RF and GNNs was reduced with the inclusion of the proposed loss. Therefore, results on this benchmark data set support the use of the new loss function for more consistent explanations in cases where GNN is the preferred modeling approach, e.g. for data sets where GNNs’ predictive performance is superior to RF.

Along those lines, and as a potential caveat, during our experiments we had noticed that the explainability improvement provided by the UCN loss seemed to be dependent on the choice of GNN architecture and its associated predictive performance, albeit the reasons for this dependency remain a topic for further study. As a general rule of thumb, we recommend that careful predictive benchmarking is performed on a case-by-case scenario before using the proposed UCN loss for interpretability.

The requirement of precomputed common substructures between pairs of compounds might be considered a limitation of the presented method. Exact MCS algorithms are computationally expensive, but the issue may be bypassed using approximations or matched molecular pair analyses [49, 50]. As ventures for future research, the exploration of additional GNN architectures and the effect on explainability might be beneficial. Herein, UCN loss has shown to be successful for a specific architecture which has become standard in the field [4]. Moreover, feature attribution approaches may be hindered by some of the current GNN training limitations. Other promising topics for future investigations might include exploring architectures that avoid the Weisfeler-Lehman graph isomorphism issue, or tackling the oversmoothing effect on GNNs [51] by applying regularization [52, 53], self-supervised learning [54, 55], or pretraining techniques [56]. All in all, a new strategy for GNN explainability was introduced, inspired by the lead optimization efforts in drug discovery, which are centered on specific chemical series. The presented explainability approach has to potential to help rationalizing GNN-based model decisions in that context.

## Supplementary Information

**Additional file 1.** Global direction results on training and test sets, color agreement metrics on all sets, neural network training hyperparameters, and feature attribution techniques settings, are reported in the Additional file to this manuscript.

## Data Availability

Code to replicate the results in this paper is provided in https://github.com/microsoft/molucn, and distributed under a permissive MIT license. All associated data, results and training logs are also provided.
