# Supplementary material for: Explaining compound activity predictions with a substructure-aware loss for graph neural networks
Source: J Cheminform. 2023 Jul 25;15:67. doi: 10.1186/s13321-023-00733-9 (PMC10369817; doi:10.1186/s13321-023-00733-9)
Supplement: Supplementary file 1 — Additional file 1. Global direction results on training and test sets, color agreement metrics on all sets, neural network training hyperparameters, and feature attribution techniques settings, are reported in the Additional file to this manuscript. [file 13321_2023_733_MOESM1_ESM.pdf]

Explaining compound activity predictions with a substructure-aware loss for graph neural networks
Supporting information

Kenza Amara,[†,‡] Raquel Rodríguez-Pérez,[*,¶] and José Jiménez-Luna[*,†]

†*Microsoft Research Cambridge, CB1 2FB Cambridge, United Kingdom*

‡*Department of Computer Science, ETH Zurich, 8092 Zurich, Switzerland*

¶*Novartis Institutes for Biomedical Research, 4002 Basel, Switzerland*

E-mail: raquel.rodriguez_perez@novartis.com; jjimenezluna@microsoft.com

List of Tables

S1 Node and edge molecular graph features used in the training of the GNN models 8

S2 Architectural details of the GNN models used throughout this study and additional training hyperparameters 8

List of Figures

S1 Performance differences between GNN (MSE+UCN loss) and RF models at varying training set size. Root mean squared error (RMSE) differences between a GNN model trained with the MSE+UCN loss fucntion and a RF model are reported for targets with increasing numbers of training pairs. The number of training pairs per target was binned considering the quantiles (Q1-Q4) of the distribution. For targets with more training pairs, performance differences between the methods decreased. 3

S2 Absolute difference of global direction at varying scaffold size and across feature attribution methods. (a) Global direction and (b) weighted global direction values are reported at different thresholds of minimum shared MCS among testing pairs (%). In (b), global direction is weighted by the number of pairs per each target. Results are shown for the difference of two loss functions, *i.e.* $\mathcal{L}_{\text{MSE+UCN}} - \mathcal{L}_{\text{MSE}}$ (left panels) and $\mathcal{L}_{\text{MSE+UCN}} - \mathcal{L}_{\text{MSE+AC}}$ (right panels). Colors report different feature attribution methods, five for GNN models and atom masking for RF models. 4

S3 Target-specific global direction at the 50% MCS threshold for all molecular test pairs, for both the MSE and the hereby-proposed UCN loss.Compound pairs considered at the minimum 50% MCS threshold. 5

S4 Global direction at different MCS thresholds for training set molecules. Results are reported for different feature attributions and loss functions. 5

S5 (a) Color agreement and (b) weighted color agreement accuracy at different MCS thresholds for the molecules in the test set. Results are reported for different feature attributions and loss functions. 6

S6 (a) Color agreement and, (b) weighted color agreement accuracy at different MCS thresholds for the molecules present in the train pairs and for the different loss strategies evaluated. 7

1. Disaggregated predictive performance

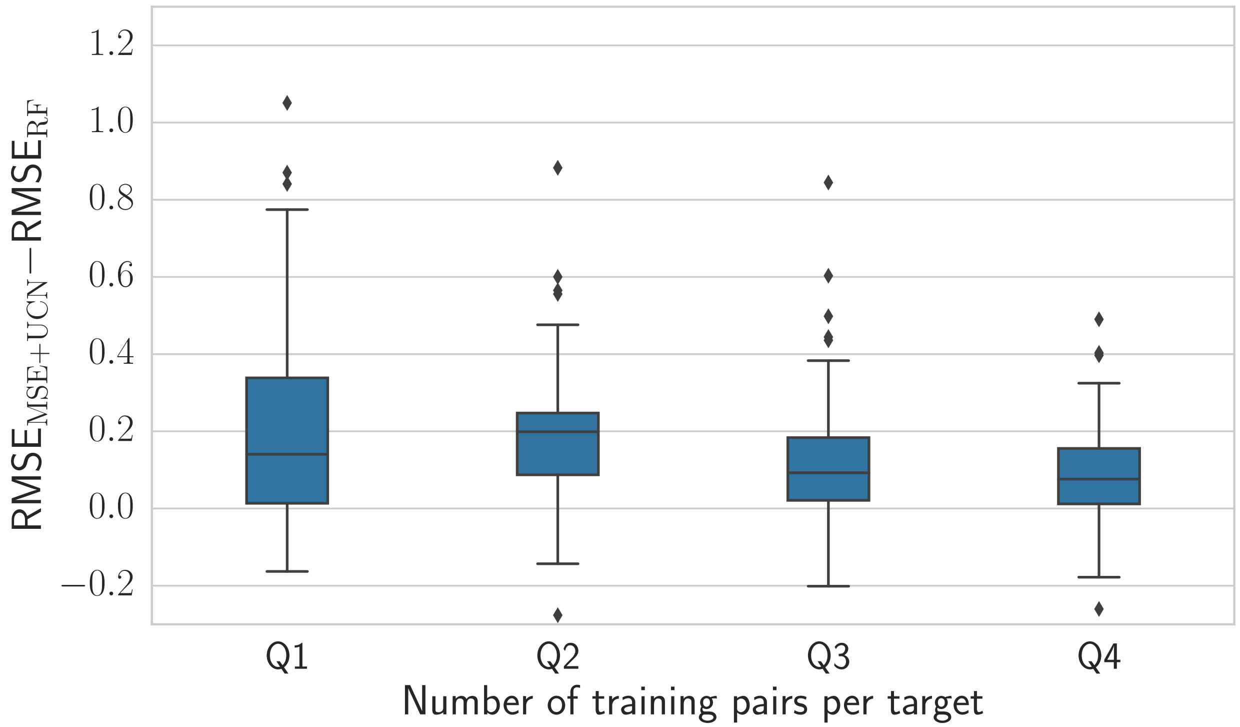

Figure S1: Performance differences between GNN (MSE+UCN loss) and RF models at varying training set size. Root mean squared error (RMSE) differences between a GNN model trained with the MSE+UCN loss fucntion and a RF model are reported for targets with increasing numbers of training pairs. The number of training pairs per target was binned considering the quantiles (Q1-Q4) of the distribution. For targets with more training pairs, performance differences between the methods decreased.

2. Additional global direction results on the test sets

Figure S2 shows the absolute difference in global direction (and its weighted version) between $\mathcal{L}_{\text{MSE+UCN}} - \mathcal{L}_{\text{MSE}}$ and $\mathcal{L}_{\text{MSE+UCN}} - \mathcal{L}_{\text{MSE+AC}}$.

Figure S3 reports the absolute number of protein targets with an improvement in the global direction metric upon the inclusion of the proposed UCN loss. A high proportion of targets showed improved performance with the UCN loss compared to the standard MSE

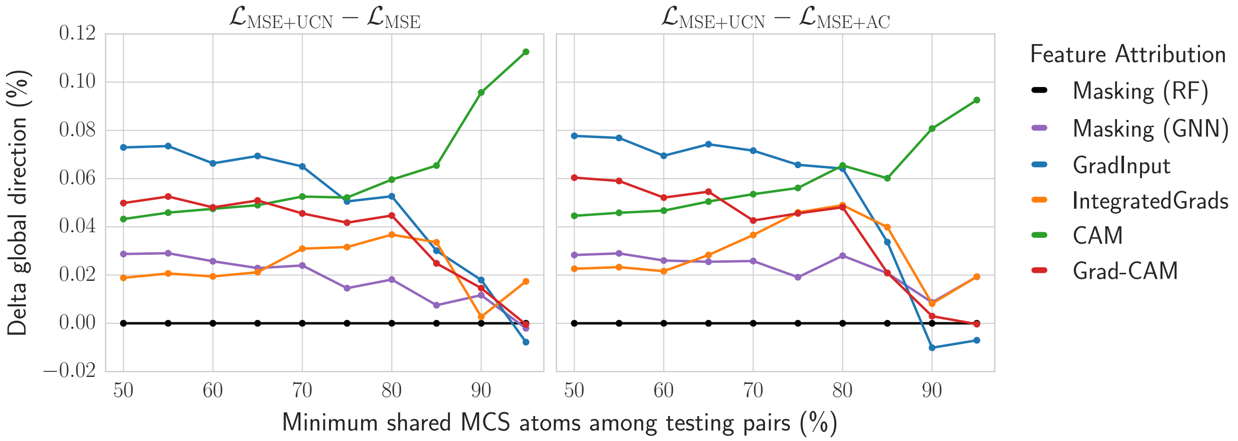

(a)

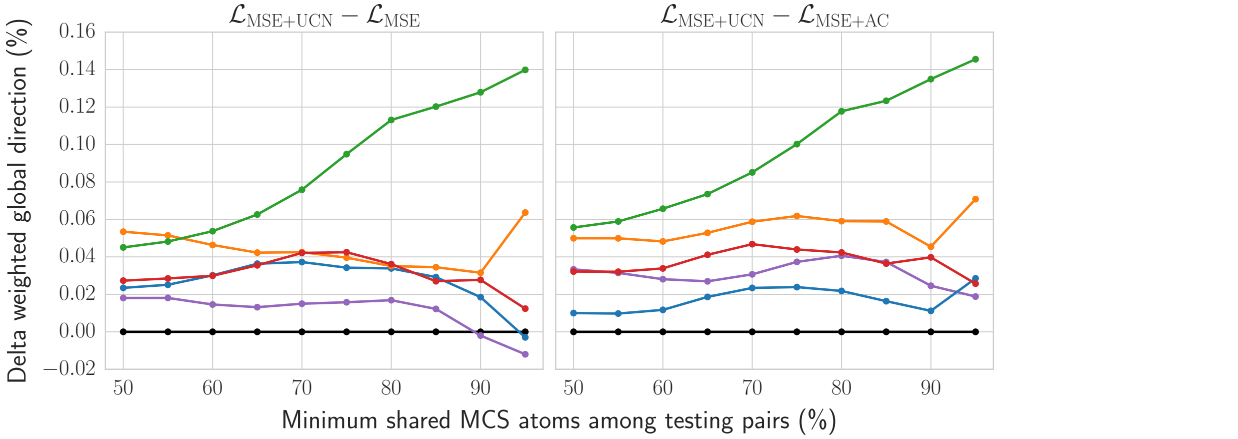

(b)

Figure S2: Absolute difference of global direction at varying scaffold size and across feature attribution methods. (a) Global direction and (b) weighted global direction values are reported at different thresholds of minimum shared MCS among testing pairs (%). In (b), global direction is weighted by the number of pairs per each target. Results are shown for the difference of two loss functions, *i.e.* $\mathcal{L}_{\text{MSE+UCN}} - \mathcal{L}_{\text{MSE}}$ (left panels) and $\mathcal{L}_{\text{MSE+UCN}} - \mathcal{L}_{\text{MSE+AC}}$ (right panels). Colors report different feature attribution methods, five for GNN models and atom masking for RF models.

loss.

3. Global direction on the training sets

Global direction results for the training sets are reported in Figure S4. In line with the results reported on the main manuscript, inclusion of the UCN loss yielded an improvement

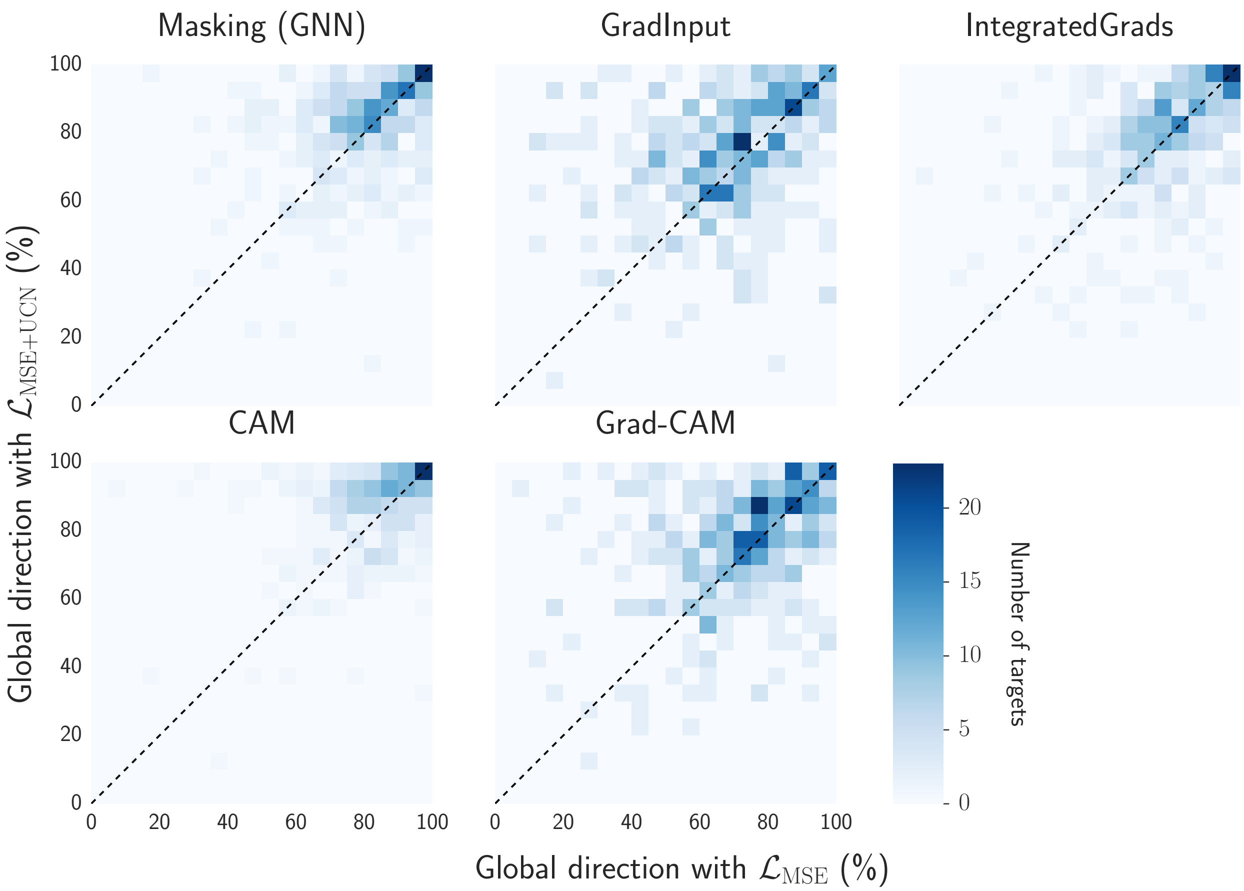

Figure S3: Target-specific global direction at the 50% MCS threshold for all molecular test pairs, for both the MSE and the hereby-proposed UCN loss.Compound pairs considered at the minimum 50% MCS threshold.

in global direction for most of the methods.

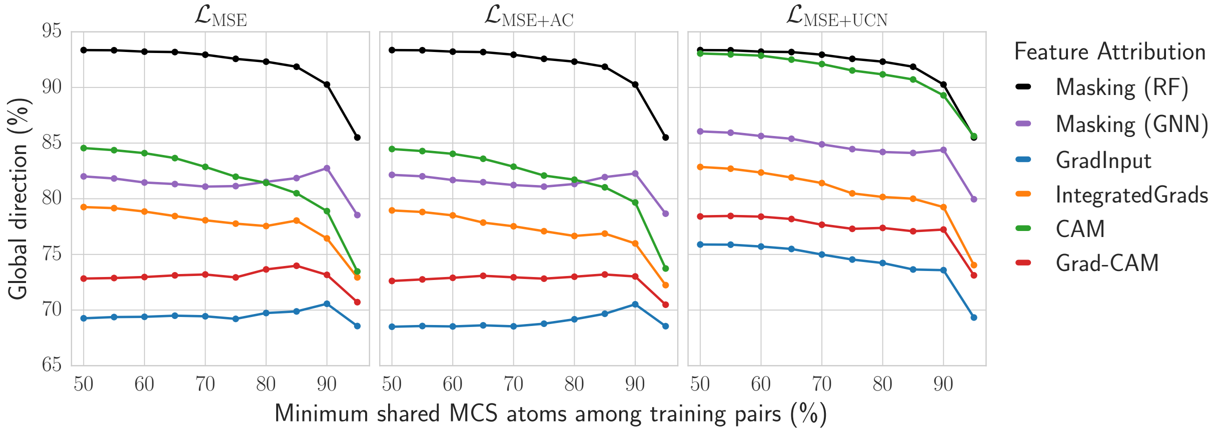

Figure S4: Global direction at different MCS thresholds for training set molecules. Results are reported for different feature attributions and loss functions.

4. Color agreement

Figures S5 and S6 report color agreement accuracy for the training and test sets, respectively. Similar conclusions as those presented in the main results section can be drawn, with the UCN loss improving results for several of the feature attribution methods evaluated. For this metric, the advantage is less pronounced.

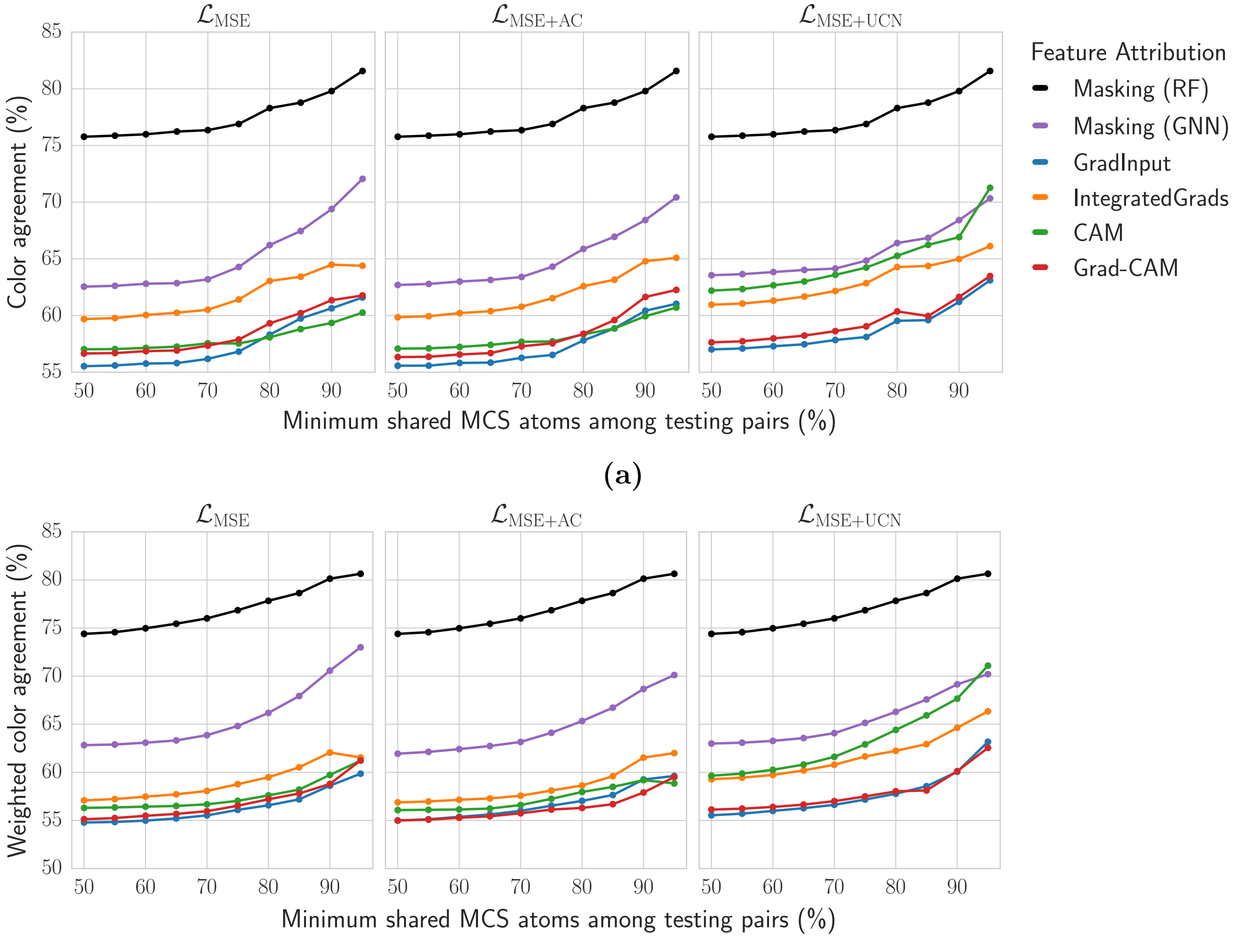

(a)

(b)

Figure S5: (a) Color agreement and (b) weighted color agreement accuracy at different MCS thresholds for the molecules in the test set. Results are reported for different feature attributions and loss functions.

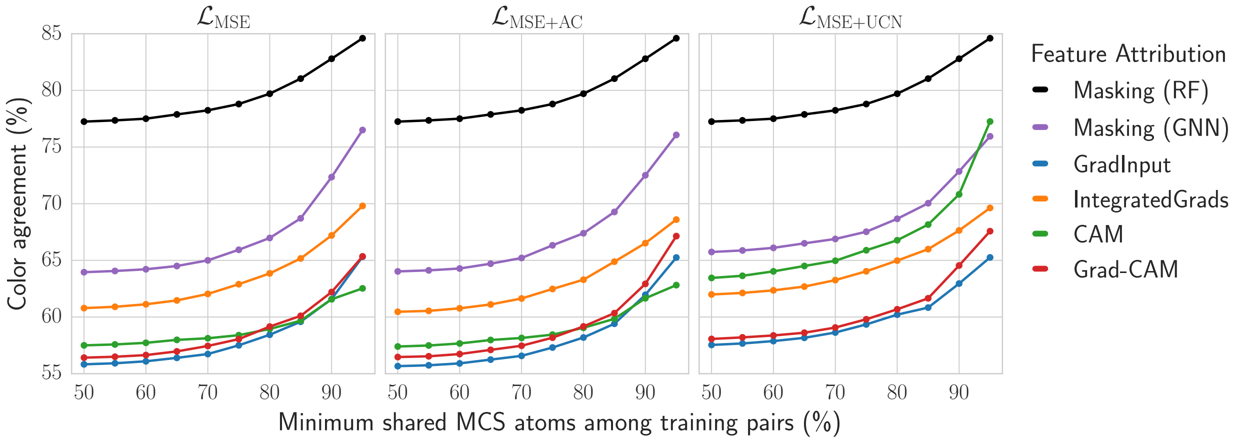

(a)

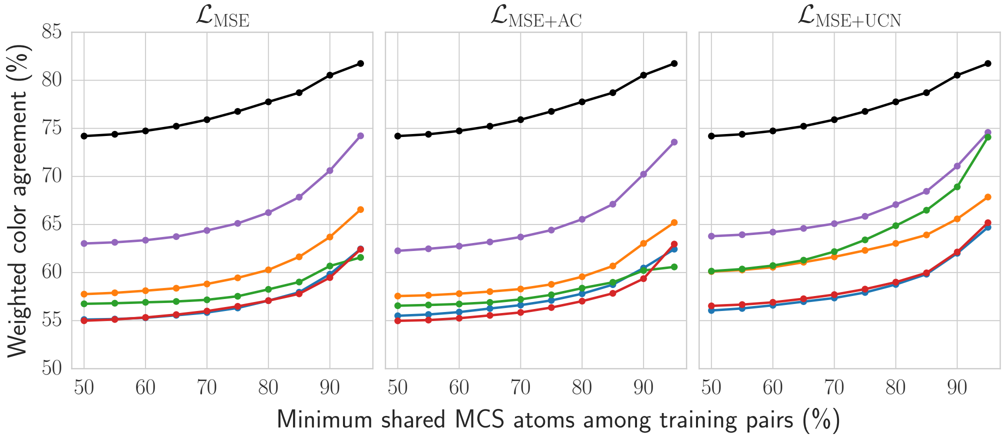

(b)

Figure S6: (a) Color agreement and, (b) weighted color agreement accuracy at different MCS thresholds for the molecules present in the train pairs and for the different loss strategies evaluated.

5. Neural network hyperparameters and featurization

The trained GNN models featured 2 message-passing layers over the bonds of the molecular graph, each with 32 neurons. Graphs were featurized with the descriptors provided in Table S1. A mean node pooling strategy was used for all losses considered in the manuscript, followed by two multi-layer perceptron modules with 32 and 16 hidden nodes, respectively and with ReLU activation functions. In the case of the CAM feature attribution method, only 1 multilayer perceptron with 32 hidden nodes was used to adhere with GNN node latent size. GNN and other training-related hyperparameters are described in Table S2. All

GNN models were trained using the PyTorch geometric software package[1]. RF models consisted of 1000 base tree learners and used 1024-bit binary Morgan fingerprints as molecular descriptors[2]. For RF, the scikit-learn implementation[3] was utilized.

Table S1: Node and edge molecular graph features used in the training of the GNN models

**Description level**	**Features**
Atom	atom type, number of heavy atom neighbors, formal charge, hybridization, presence in ring, aromaticity, atomic mass, van der Waals radius, covalent radius
Bond	bond type, bond stereo, conjugation, presence in ring

Table S2: Architectural details of the GNN models used throughout this study and additional training hyperparameters

**Model**		**Training**	
Base layer	NNConv	Batch size	16
# layers	2	Optimizer	Adam
Hidden Dim.	32	# epochs	200
Readout f.	mean	Learning rate	10^{-3}

6. Additional details on the feature attribution techniques

A brief technical description of each feature attribution method used throughout this work is now provided. We refer the reader to the accompanying repository of this work for further details. We denote a message-passing model as a learnable function that maps graphs to arbitrary target values $f_\theta : (\mathcal{G}, \mathcal{X}) \rightarrow \mathcal{Y}$. A feature attribution approach in this context takes a graph with featured vertices and edges and produces an importance score $\mathcal{E} : \mathcal{G} \rightarrow c_v, b_{u,v}$, for each $u, v \in \mathcal{G}$. Using this notation, the used feature attribution methods throughout this study are detailed below:

- **GradInput**: Gradient backpropagation from the predicted target is performed onto the input features of the nodes and edges of the graph $\left(i.e. \frac{\partial f_\theta}{\partial x_v}, \frac{\partial f_\theta}{\partial w_e}\right)$. This gradient is then elementwise multiplied by the respective input to produce a final importance value.

- **Integrated Gradients** was proposed as an theoretically grounded alternative to Grad-Input that avoids several limitations, such as graph saturation, while guaranteeing model sensitivity and implementation invariance. This approach aggregates the gradient of the predicted target with respect to the (node) input features through a path integral starting from a user-defined baseline x'_v:

$$\mathrm{IG}(x_v) = (x_v - x'_v) \int_\Omega \frac{\partial f\left(x'_v + \alpha\left(x_v - x'_v\right)\right)}{\partial x_v} d\alpha, \tag{1}$$

  The same can be applied to the edge inputs w_e. Since this integral is not tractable, Riemann approximations are in practice computed by sampling uniformly across α.

- **Class Activation Maps (CAM)** assumes that the model uses a global average pooling layer before prediction and decomposes the final output as a linear sum of node and edge activations. Specifically, for graphs, the product between activations and the weights of the last linear layer after the readout operation is taken. Overall, the importance for a single node feature is given by

$$\mathrm{CAM}(x_v) = \mathrm{GAP}_\theta(G_v)\omega_\theta(G_v), \tag{2}$$

  where CAM and ω represent the GAP activations and weights of the last linear layer after graph readout, respectively.

- **GradCAM** is a gradient-based variant of the CAM method that uses the gradient of the predicted value to remove the necessity of a GAP layer. Specifically, the intermediate activations multiplied by the output gradient is used as a measure of importance:

$$\text{GradCAM}(x_v) = \frac{1}{K} \sum_k a_{k,\theta}(G_v) \partial_{k,\theta}(G_v), \tag{3}$$

where $k \in K$ are the number of intermediate layers, a are the intermediate layer activations and ∂ is the gradient of the prediction w.r.t. each of these activations.

Feature attribution was computed for both edges and nodes of the input graph. Similar to prior studies[4], edge attributions were then halved and equally distributed to the nodes connected by the edge. For the Monte Carlo approximation of the path integral in the Integrated Gradients method, 50 steps were used.

References

(1) Fey, M.; Lenssen, J. E. Fast graph representation learning with PyTorch Geometric. *arXiv preprint arXiv:1903.02428* **2019**,

(2) Rogers, D.; Hahn, M. Extended-connectivity fingerprints. *J. Chem. Inf. Model.* **2010**, *50*, 742–754.

(3) Pedregosa, F.; Varoquaux, G.; Gramfort, A.; Michel, V.; Thirion, B.; Grisel, O.; Blondel, M.; Prettenhofer, P.; Weiss, R.; Dubourg, V., et al. Scikit-learn: Machine learning in Python. *J. Mach. Learn. Res.* **2011**, *12*, 2825–2830.

(4) McCloskey, K.; Taly, A.; Monti, F.; Brenner, M. P.; Colwell, L. J. Using attribution to decode binding mechanism in neural network models for chemistry. *Proc. Natl. Acad. Sci. U.S.A.* **2019**, *116*, 11624–11629.
